# AMI, an Indazole Derivative, Improves Parkinson’s Disease by Inhibiting Tau Phosphorylation

**DOI:** 10.3389/fnmol.2020.00165

**Published:** 2020-11-19

**Authors:** Zhang Mao, Zhu Wen-ting, Wang Hai-tao, Yu Hui, Lan Shi-yi, Xu Jiang-ping, Wang Wen-ya

**Affiliations:** ^1^School of Pharmaceutical Sciences, Southern Medical University, Guangzhou, China; ^2^The Third Affiliated Hospital of Guangzhou Medical University, Guangzhou, China; ^3^School of Basic Medical Science, Southern Medical University, Guangzhou, China.

**Keywords:** 6-amino-1-methyl-indazole, Parkinson’s disease, tau, SH-SY5Y, MPTP

## Abstract

Dopaminergic neuronal loss is the main pathological character of Parkinson’s disease (PD). Abnormal tau hyperphosphorylation will lead to dopaminergic neuronal loss. An indazole derivative 6-amino-1-methyl-indazole (AMI) successfully synthesized to inhibit tau hyperphosphorylation may exert a neuroprotective effect. The *in vitro* study showed that AMI effectively increased cell viability and alleviated the apoptosis induced by MPP^+^ in SH-SY5Y cells. In addition, AMI treatment significantly decreased the expression of p-tau and upstream kinases GSK-3β. In the MPTP-induced PD mice models, we found AMI apparently preserved dopaminergic neurons in the substantia nigra and improved the PD behavioral symptoms. Our results demonstrate that AMI exerts a neuroprotective effect by inhibiting tau hyperphosphorylation, representing a promising new candidate for PD treatment.

## Introduction

Parkinson’s disease (PD) is a common neurodegenerative disease. The apoptosis of substantia nigra dopaminergic neurons plays a pivotal role in the pathogenesis of PD (Del Rey et al., 2018). The Lewy bodies or neurites (Power et al., 2017; Liu et al., 2019a) was found in the advanced PD. Therefore, we investigate the signaling pathways that regulate neuronal apoptosis as a novel therapeutic target of PD.

Tau protein is an important microtubule-associated protein in the central nervous system, which mainly regulate by phosphorylation. Its induces and promotes the aggregation of microtubules (Lee et al., 2019). Hyperphosphorylated tau interacts with α-synuclein to promote aggregation and fibrosis, causing the formation of Lewy bodies and dysfunction of axonal transport (Singh et al., 2019). Moreover, hyperphosphorylation and aggregation result in the formation of paired helical filaments (PHFs; Zhou et al., 2018). Taltirelin, a long-acting TRH analog, down-regulated the levels of p-tau (S396), exhibiting the neuroprotective effect in both cellular and animal models of PD (Zheng et al., 2018). Formulated Chinese Medicine Shaoyao Gancao Tang reduces tau aggregation and exerts neuroprotection (Chen et al., 2018). Together, phosphorylation of tau protein mediates the pathological process of Alzheimer’s disease (AD) or PD, which is a potential therapeutic target treatment for neurodegenerative disease (Winer et al., 2018; Chen et al., 2019).

Indazole derivatives have multi-pharmacological activities such as anti-inflammatory, antibacterial and anti-tumor activities (Denya et al., 2018; Liu et al., 2019b). Our previous studies have found that 6-hydroxy-1H-indazole, 5-hydroxy-1H-indazole and 6-nitro-1H-indazole inhibit the phosphorylation of tau protein, and have protective effects on MPP^+^-induced apoptosis of SH-SY5Y cells. Particularly, 6-hydroxy-1H-indazole has shown a neuroprotective effect on 100 μm MPP^+^-induced apoptosis of SH-SY5Y cells (Liang et al., 2016). In this study, 6-nitro-1H-indazole was used as the mother nucleus to further optimize the drug structure, and to explore the indazole derivatives, which can specifically inhibit the phosphorylation of tau protein. Since the drug needs to enter the brain to treat PD, our group introduced a 1-position of methyl to 6-nitro-1H-indazole for increasing the liposoluble of the compound, and finally the novel small molecule 6-amino-1-methyl-indazole (AMI) was obtained.

In this study, we examined the effect of AMI in a PD model. MTT assays and Hoechst 33258 staining were used to assess the protective effect of AMI on SH-SY5Y cells after MPP^+^ treatment. Meanwhile, MPTP-induced PD animal models were used to detect the effects of AMI *in vivo*. The levodopa (L-DOPA) is the first-line treatment of PD, we chose L-DOPA as a control drug in our experiments.

## Materials and Methods

### Material and Antibody

Methyl-4-phenylpyridine (MPP^+^; #D048), 1-methyl-4-phenyl-1,2,3,6-tetrahydro-pyridine (MPTP; #M0896) and levodopa (#D9628) were obtained from Sigma (St. Louis, MO, USA). An ABC reagent Box (Vector PK-6101 Rabbit IgG) and Golgi staining kit (PK401) were obtained from FD NeuroTechnologes (Columbia, MD, USA). P-tau (Ser396; #ab109390) and t-tau (#ab32057) were purchased from Abcam (Cambridge, MA), while GSK-3β (#12456) and phosphorylated GSK-3β (p-GSK-3β, ser9, #9323) were purchased from Cell Signaling Technology (Danvers, MA, USA). Anti-tyrosine hydroxylase (TH) was obtained from Santa Cruz (Dallas, TX, USA). Immoblilon PVDF membranes (#ISEQ00010) and Immobilon Western Chemiluminescent HRP Substrate (#WBKLS0100) were purchased from Merck Co. (Darmstadt, Germany).

SPF grade C57BL/6 mice, male 6–7 weeks, weight 22–27 g, were purchased from Guangdong Experimental Animal Center, license number: SYXK (Yue) 2016–0167, then free drinking water, feeding to 10–11 weeks. All of the experimental protocols were approved by the Institutional Animal Care and Use Committee of Southern Medical University (Guangzhou, Guangdong, China).

### Synthesis of AMI

6-nitro-1H-indazole was dissolved in dimethylformamide and then methyl iodide and sodium cyanide were added according to the molar ratio of 6-nitro-1H-indazole: iodomethane: sodium hydride = 1:2:2. The reaction was stirred for 24 h, then filtered and extracted three times with ethyl acetate, dried over anhydrous sodium sulfate, and evaporated to produce 6-nitro-1-methyl-indazole. Subsequently, 6-nitro-1-methyl-indazole was dissolved in methanol, palladium on carbon was added as a catalyst, the reaction of hydrogen through 4 h, and finally filtered and separated by column chromatography to obtain AMI.

### MTT

MTT assay was used to determine the cell viability for SH-SY5Y cells. The SH-SY5Y cells were seeded at a density of 1 × 10^4^ cells/well in 96-well plates and incubated overnight. Firstly, different concentrations of MPP^+^ acts on SH-SY5Y cells, and confirm the optimal concentration of MPP^+^ on the cells. The addition of MPP^+^ to SH-SY5Y cultures was performed at 300 μM. Afterwards, the cells were pretreated with different concentrations of AMI for 2 h, then added with MPP^+^ at a final concentration of 300 μM for 48 h. Add 100 μl of 0.5 mg/ml MTT working solution to each well, and incubate for 4 h at 5% CO_2_ at 37°C. The supernatant was discarded, each well was added 100 μl DMSO, and shake at low speed for 10 min to fully dissolve the crystals. The absorbance of each group of samples was measured with a microplate reader (Bio-Rad Model 680) at a wavelength of 570 nm. Untreated cells represented 100% viability.

### Hoechst 33258 Staining

The SH-SY5Y cell line was pretreated with AMI for 2 h, and then MPP^+^-induced apoptosis for 48 h. The cells were fixed 10 min with fixative. After the fixative was removed, it was washed twice with PBS for 3 min each time and then 0.5 ml of Hoechst 33258 staining solution was added, staining the cell line for 5 min. After the staining solution was removed, the culture solution was washed twice with PBS for 3 min. A drop of anti-fluorescence quenching sealant was applied to the slide. The slide was covered with a cell-coated cover glass, and blue nuclei were detected by a fluorescence microscope. The excitation wavelength was about 350 nm and the emission wavelength was about 460 nm.

### Western Blot Analysis

Western blott was used to determine the expression of GSK-3β, p-GSK-3β(ser9), Tau, p-Tau(ser396) and TH. After drug treatment, cells or tissues were quickly collected and lysed with RIPA buffer containing 1% protease and 1% phosphatase inhibitor, and shaken on ice for 40 min. The lysate was then centrifuged at 12,000 rpm for 20 min at 4°C and 10 min at 12,000 rpm. The supernatant was collected and the protein concentration was determined using a BCA protein assay kit. An equal amount of protein was separated by SDS-polyacrylamide gel electrophoresis and transferred to a 0.4 μm pore size hydrophilic PVDF membrane. Non-specific binding was blocked with 5% w/v BSA, and membranes were then incubated in milk dissolved in TBST for 2 h at room temperature. The membrane was washed three times with TBST for 10 min, and then incubated with primary antibodies at 4°C overnight. After rinsing and incubation with secondary antibodies, the blot signals were detected with a Bio-Rad Ultraviolet Imaging System, and immunoblotting was quantified with ImageJ software.

### Parkinson Animal Model Preparation and Administration

The C57BL/6 male mice were divided into five groups (12 in each group). The control group received normal saline. The PD model group was treated with 30 mg/kg MPTP(dissolved in normal saline) by intraperitoneal (i.p.) injection daily for 5 consecutive days (Schildknecht et al., 2017). Mice were treated with AMI (2 and 4 mg/kg/day, dissolved in normal saline containing 3% DMSO) half an hour before MPTP treatment. The L-DOPA group was given 10 mg/kg/day (dissolved in normal saline containing 3% DMSO + 0.5% CMC-Na, i.p.), 2 h after MPTP treatment. Meanwhile, we recorded the incubation period and duration of the mice after administration. The latent period began with intraperitoneal injections of MPTP into mice until paralysis occurred. Ten days after the final injection of MPTP, the mice conducted behavioral experiments and then sacrificed (Figure 3A).

**Figure 1:**
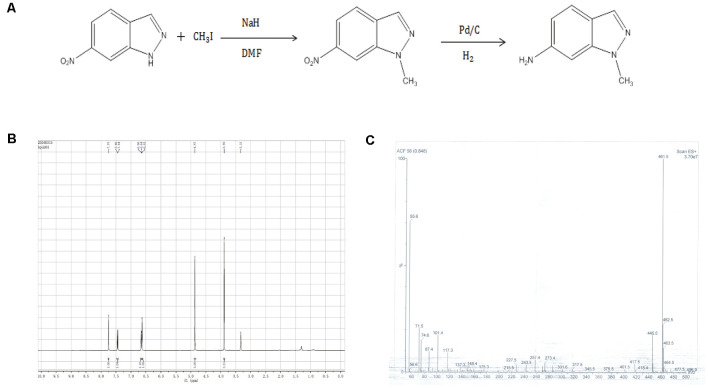
**(A)** Synthetic route 6-amino-1-methyl-indazole (AMI). **(B)** AMI hydrogen spectrum. **(C)** AMI mass spectrum.

**Figure 2:**
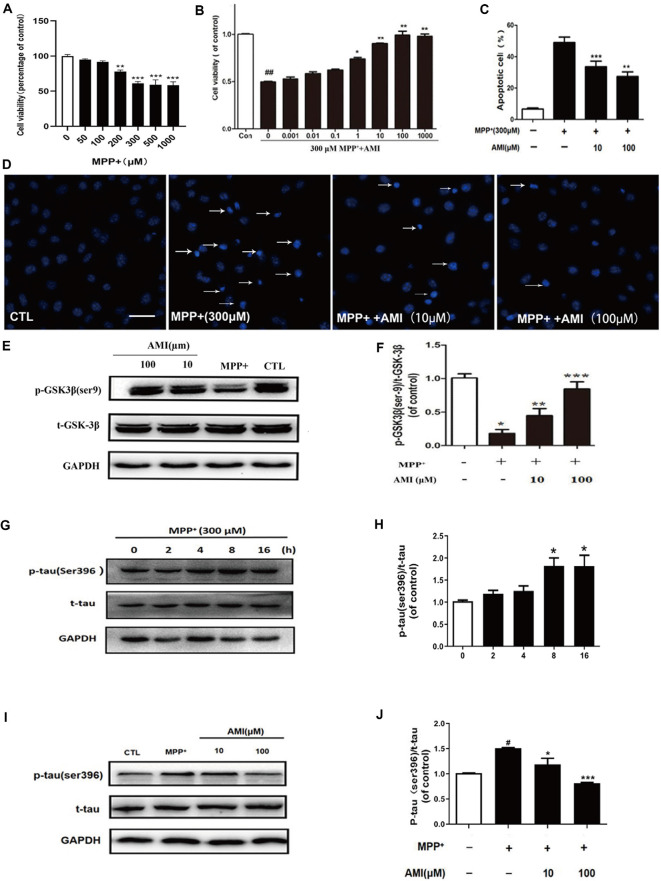
AMI inhibits apoptosis of SH-SY5Y cells induced by MPP^+^. **(A)** The MTT assay was performed to assess the effect of MPP^+^ on the proliferation of SH-SY5Y cells. **(B)** The MTT assay was performed to assess the effect of AMI on the proliferation of SH-SY5Y cells. Representative images **(D)** and quantitative data **(C)** of Hoechst 33258 nuclear staining in SH-SY5Y cells, bar = 50 μM. **(E)** AMI increased intracellular p-GSK-3β (Ser9) levels in SH-SY5Y cells treated with MPP^+^. **(F)** Densitometric quantification of p-GSK3β (ser-9)/t-GSK-3β protein levels. **(G)** MPP^+^ increased intracellular p-tau (Ser396) levels in SH-SY5Y cells. **(H)** Densitometric quantification of p-tau (ser396)/t-tau protein levels in different time. **(I)** AMI decreased intracellular p-tau (ser396)/t-tau protein levels at 8 h. **(J)** Densitometric quantification of p-tau(ser396)/t-tau protein levels at 8 h after treating with AMI. *p < 0.05, **p < 0.01, ***p < 0.001 vs. MPP^+^ group. ^#^*p* < 0.05, ^##^*p* < 0.01 vs. vehicle group (control).

**Figure 3:**
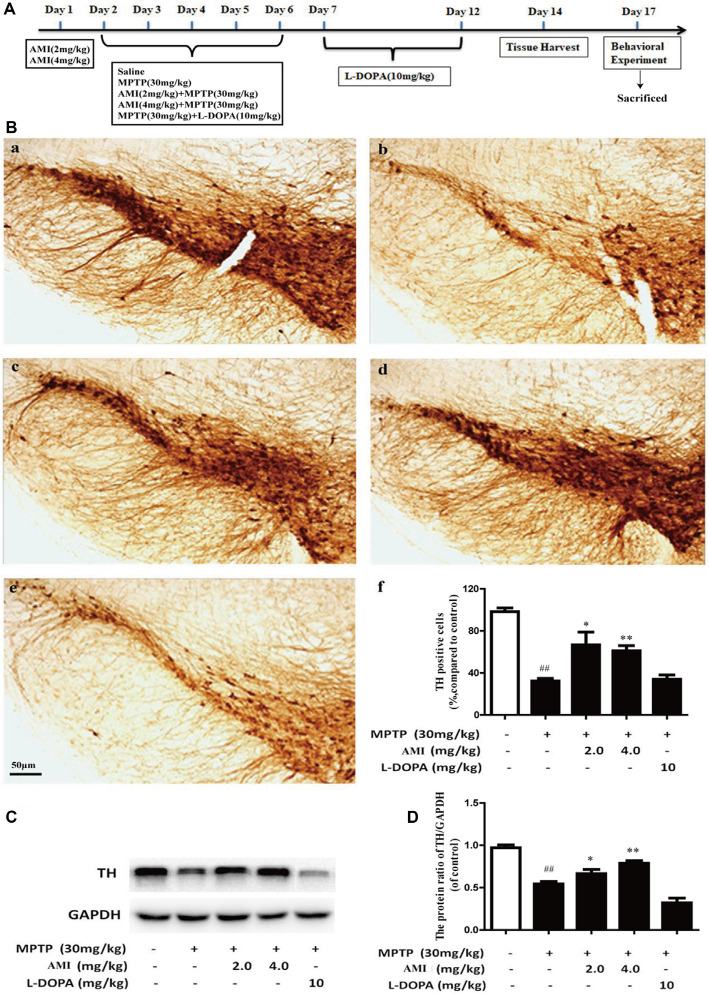
**(A)** Experimental dosing regimen. **(B)** Representative images and quantitative data of TH staining in the substantia nigra of midbrain. Magnification: 40× (a, Control; b, MPTP; c, AMI 2 mg/kg; d, AMI 4 mg/kg; e, L-DOPA 10 mg/kg; f, the number of TH immunopositive cells in the substantia nigra). **(C)** TH protein expression was examined by western blot analysis. **(D)** The protein levels of TH were quantified by densitometry. Slice thickness: 35 μm. ^##^*p* < 0.01 vs. control; *p < 0.01, **p < 0.001 vs. MPTP.

### TH Immunohistochemistry

Seven days after the last injection of MPTP, the ventral midbrain was isolated and the TH content was detected by immunohistochemistry. Brain slices (35 μm) were rinsed with 0.3% Triton for 30 min, and then treated with freshly prepared 3% H_2_O_2_ for 1 h. The brain slices were rinsed twice in PBS for 10 min each, and rinsed for 1 h with serum, then incubated overnight with rabbit anti-TH (1:10,000, 3%BSA with PSB) at 4°C. Brain slices were then washed three times with PBS for 10 min at room temperature and incubated with the secondary antibody of the kit for 1 h at room temperature. Next, brain slices were incubated for 1 h in ABC kit (Vector PK-6101 Rabbit IgG) and washed three times with PBS. Finally, the color was developed in DAB for 2–5 min, the brain slices were rinsed with PBS, and the slices were dried. After the patches were dehydrated and transparent, they were photographed under a light microscope at a magnification of ×400. The numbers of TH-positive dopaminergic neurons in the substantia nigra of each mouse were counted. The mean number of right and left sides was regarded as the neuronal cell number of each mouse (Wang et al., 2007).

### Golgi Staining

To study the morphological changes of nerve dendrites and dendritic spines in animal brains after drug treatment by Golgi staining (Zhong et al., 2019). Seven days after the last injection of MPTP, the substantia nigra slices were obtained for Golgi staining. According to the Golgi staining (FD Neuro Technologes) manufactures’manual, we mixed liquid A and liquid B for 24 h and stored it in the dark. The whole brain placed into the prepared staining solution in the dark for 3 weeks at normal temperature, and transferred to solution C kept in the dark for at least 72 h. Then midbrain was fixed with an embedding solution, sliced on a frozen slicer (100 μm), attached to a glass slide, immersed in solution C, and dried for sectioning. The sections were washed twice with double distilled water for 4 min each time. The sections were then incubated for 10 min in a working solution consisting of solution D, solution E and double distilled water = 1:1:2. Neuronal morphology was observed under a microscope.

For the analysis of dendritic branches, non-axonal synapses longer than 10 μm and directly from the cell body are defined as primary dendrites. All protrusions that emerge from the primary tree are called secondary dendrites, and all middle-end protrusions below 10 μm are called axonal tips. When performing a sholl analysis, multiple concentric circles centered on the cell body that differ in diameter by 15 μm from each other, and all protrusions in the concentric circles are counted.

### Behavioral Experiment

Pole-climbin, rotarod test and traction test were performed 10 days after the final injection of MPTP (Wang et al., 2007). There are 6–8 mice per group, all behavioral tests were conducted in a double-blinded manner.

In traction test, the traction test measures muscle strength. A cord with a diameter of 0.5 cm was placed 70 cm horizontally. Each mouse’s front paws were hung on the rope and the mouse was released. The time of the fall was recorded, as was whether the mouse pulled its hind limbs to the rope. The feces and urine of the mice were washed off before the experiment.

In rotarod test, a rotarod test is a common method to assess neuromuscular coordination. First, mice were positioned on a rotating rod (6 cm diameter) for 30 s, and then trained at a constant speed of 12 rpm for 180 s. Sixty minutes after the last training, a mouse was placed on the rod and the incubation period of its fall was recorded as the end point measurement. The average time of three trials was calculated for statistical analysis.

In pole-climbing test, Mice were placed near the top of rough surface wooden stick (10 mm in diameter and 55 cm in height) and their heads facing up. The total time for each animal to reach the floor was recorded, with a maximum duration of 120 s. Each mouse was tested three times at 10 min intervals and the average time was calculated for statistical analysis.

### Statistical Analysis

Statistical analyses were performed using SPSS 13.0. Results were expressed as mean ± SD. One-way ANOVA was employed for multiple group comparisons. The statistical significance was set at *P* < 0.05.

## Result

### Characterization of AMI

AMI was obtained by a two-step reaction (Figure 1A), and the hydrogen spectrum (Figure 1B) and mass spectrum (Figure 1C) shown a characteristic absorption peak as the AMI.

### AMI Improves Apoptosis in SH-SY5Y Cells After MPP^+^ Treatment

The *in vitro* cytotoxicity of MPP^+^ against human SH-SY5Y cells was by using the MTT assay. As shown in the results of Figure 2A, MPP^+^ can inhibit the growth of human SH-SY5Y cells in a dose-dependent manner. We chose 300 μM MPP^+^ for cell experiment. SH-SY5Y cells were pretreated with 10 and 100 μM AMI for 2 h, and treated with 300 μM MPP^+^ for 48 h. The MTT assay indicated that AMI attenuating the cytotoxicity of MPP^+^ of SH-SY5Y cells (Figure 2B). In addition, MPP^+^ reduced the number of TH-positive cells and caused the nuclear volume to shrink. However, nuclear pyknosis was significantly reduced with AMI pretreatment for 2 h (Figures 2C,D). These results indicate that AMI have protective intervention in MPP^+^-induced apoptosis of SH-SY5Y cells.

### AMI Inhibits MPP^+^-Induced Tau (Ser396) Hyperphosphorylation in SH-SY5Y Cells

MPP^+^-induced tau hyperphosphorylation in SH-SY5Y cells, and 300 μM MPP^+^-treated SH-SY5Y cells for 8 h, showed that the expression of p-tau (Ser396) was highest, and then gradually decreased (Figures 2E,F). Therefore, we chose 8 h detect the effect of AMI on the phosphorylation level of tau. As shown in Figures 2I,J, The 10 μM and 100 μM AMI apparently reduced the level of p-tau (Ser396) after MPP^+^ treatment, total tau expression was not affected. Furthermore, MPP^+^ treatment markedly inhibited the expression of p-GSK3β(Ser9). However, pretreatment of AMI increased the phosphorylation of GSK3β in SH-SY5Y cells (Figures 2G,H). In general, we found that AMI reducing tau hyperphosphorylation may be related to its inhibition of GSK-3β activity.

### AMI Improves Symptom in C57/BL Mice After MPTP Treatment

The symptoms of the mice were observed after administration of MPTP and AMI. The MPTP group showed symptoms of whole-body tremor, vertical hair, arched back, vertical tail, and slow movement. However, the symptoms of the AMI 2.0 mg/kg and 4.0 mg/kg groups were significantly alleviated, with only mild tremors, vertical hair, arched back, and vertical tail. At the same time, the latency from the injection of MPTP to the above symptoms and the duration of PD symptoms was observed and recorded. As shown in Table 1, the latency of PD symptoms in the AMI 2.0 mg/kg and 4.0 mg/kg groups was significantly longer than that in the MPTP group, and the duration was shortened. Additionally, the duration of PD symptoms in mice was obviously shortened, but the latency was not changed after treating with levodopa compared to the MPTP group.

**Table 1:** Effect of 6-amino-1-methyl-indazole (AMI) on the latency period and duration of the MPTP model of PD symptoms in mice (*n* = 11–12, × ±s).

Group	x	Duration(s)
Control	∞	-
MPTP	197.67 ± 22.91	1039.84 ± 99.61
AMI (2 mg/kg)	225.77 ± 24.84*	905.25 ± 58.96***
AMI (4 mg/kg)	229.79 ± 22.04**	899.75 ± 42.45***
L-DOPA	200.27 ± 16.62	951.95 ± 53.23*

*p < 0.05, **p < 0.01, ***p < 0.001 vs. MPTP.

### AMI Increases DA Neurons in C57/BL Mice After MPTP Treatment

TH is an important and specific rate-limiting enzyme for DA synthesis, which has a labeling effect on DA neurons. The greater the number of TH-positive cells, the greater the content of DA. We used immunohistochemistry and western blot to detected TH expression in substantia nigra of the midbrain. As shown in Figure 3B, the survival rate of neurons in the MPTP group obviously decreased compared to the control group, which proved that the PD animal model was successfully established. However, the number of neurons in the mice pre-administered with 2.0 mg/kg AMI (65.80 ± 12.8%, *p* < 0.001) and 4.0 mg/kg AMI (64.13 ± 5.7%, *p* < 0.001) was higher than that in the MPTP group (34.84 ± 2.7%; Figures 3B–F). Additionally, the TH positive neurons in L-DOPA group (34.86 ± 4.9%) was similar to the model group. These results indicated that AMI rescues the loss of dopamine neurons in mice and has a neuroprotective effect, while levodopa has no neuronal protective effect.

To further study whether AMI has a protective effect on DA neurons, western blot analysis used to detect TH expression in midbrain. The expression of TH protein in the MPTP group was markerly lower than that in the control group. Compared with the MPTP group, the 2 mg/kg and 4 mg/kg AMI intervention groups increased the expression of TH protein. These results further suggested that AMI can ameliorate the MPTP-induced loss of TH in the substantia nigra striatum of mice, and has a protective effect on the degeneration of DA neurons (Figures 3C,D).

### AMI Enhances Substantia Nigra Dendrites in C57/BL Mice After MPTP Treatment

**Figure 4:**
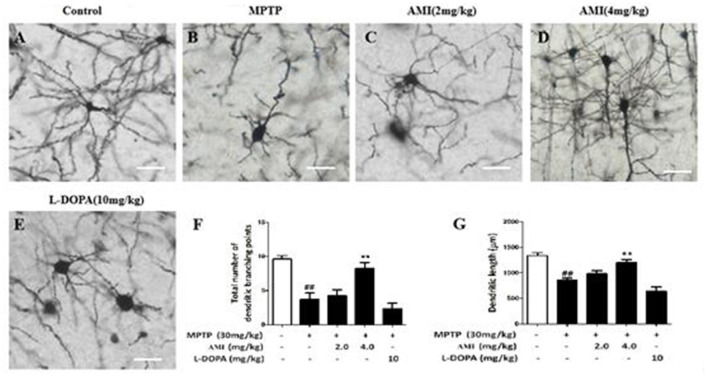
Golgi staining for detecting the density and length of neuronal dendrites in the substantia nigra of the brain **(A)** Control; **(B)** MPTP; **(C)** AMI 2 mg/kg; **(D)** AMI 4 mg/kg; **(E)** L-DOPA 10 mg/kg. **(F)** Total number of dendritic branching points. **(G)** Dendritic length. Magnification: 40×, slice thickness: 100 μm. ^##^*p* < 0.01 vs. control; **p < 0.001 vs. MPTP.

To explore the effect of AMI on the substantia nigra dendrites, golgi staining was used to detect the density and length of neuronal dendrites in the substantia nigra. As shown in Figures 4A–E, the total number of dendritic branching point in the substantia nigra of the midbrain were decreased in MPTP group (3.75 ± 0.89, *p* < 0.01) compared with the control group (9.62 ± 0.52). However, 4.0 mg/kg AMI (8.25 ± 0.89, *p* < 0.01) significantly increased the total number of dendritic branching point and dendritic length. The 2.0 mg/kg AMI (4.25 ± 0.89) group may be due to insufficient dose, demonstrating no obvious protective effect. We also found that the in L-DOPA group (2.37 ± 0.74) has a weak influence on the density of neuronal dendrites in the substantia nigra of mice (Figures 4F,G). These results indicated that AMI can effectively improve MPTP-induced dendritic spine density reduction.

### AMI Improves Behavioral Abnormalities in C57/BL Mice After MPTP Treatment

**Figure 5:**
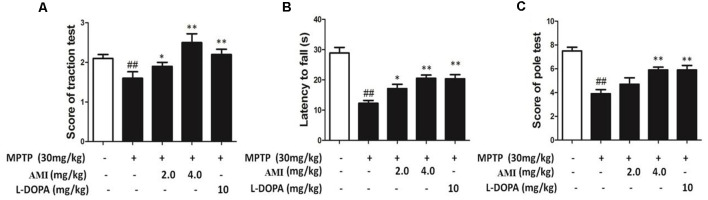
AMI improved behavioral disorders in MPTP-induced PD model mice. **(A)** Rotarod test. **(B)** Traction test. **(C)** The Pole-climbing test. ^##^*p* < 0.01 vs. control; *p < 0.01, **p < 0.001 vs. MPTP, *n* = 6–8.

We used traction test, rotarod test and pole-climbing test to investigated the behavioral ability in different treat group. As shown in Figure 5A, it was observed that the hind paws of the MPTP group were weak and the paws could not hold the wires, but the mice in other groups can hold the wire with one or two paws. The scores showed that 2.0 mg/kg AMI group (2.50 ± 0.53), 4.0 mg/kg AMI group (2.38 ± 0.52), L-DOPA group (2.71 ± 0.49) and control group (2.86 ± 0.38) had no significant difference, however the scores in MPTP group (1.40 ± 0.55, *p* < 0.001) was lower than the control group. In the rotarod test, AMI and L-DOPA can improve the MPTP induced postural agility and slow response (Figure 5B). Finally, the mice in the MPTP group stayed longer in each section of the rod, the scores of pole-climbing test results show that the scores in 2.0 mg/kg AMI group (8.0 ± 1.22), 4.0 mg/kg AMI group (7.60 ± 1.14), and L-DOPA group (7.60 ± 0.55) were higher than those in MPTP group (5.20 ± 1.64, *p* < 0.05; Figure 5C).

Collectively, these results suggested that the mice in the MPTP group had difficulty performing movements due to the weakness of the hind limbs. The AMI 2.0 mg/kg and 4.0 mg/kg groucomparisons. The statistical significanceps significantly improved the obstacle, demonstrating smoother movements and higher behavioral scores.

## Discussion

The most prominent pathological feature of PD is the loss of dopaminergic neurons in the substantia nigra. Therefore, developing a new drug that inhibits dopaminergic apoptosis may significantly improve treatment effect of PD (Yang et al., 2018a; Geibl et al., 2019). This study found that AMI has a protective effect on MPP^+^-induced apoptosis of SH-SY5Y cells, and this effect is accompanied by decreasing phosphorylation level of tau.

The abnormal phosphorylation of tau protein is mainly due to the imbalance of protein kinase and phosphatase regulation (Lee et al., 2019; Singh et al., 2019). When the kinase activity is increased and the phosphatase activity is decreased, the tau protein is hyperphosphorylated. Protein kinases include GSK-3 and Cyclin-dependent Kinase 5 (CDK5), in which GSK-3β phosphorylates multiple sites of the tau protein. Importantly, GSK-3β is one of the upstream tau kinases, which involved in the formation of tau phosphorylation and neurofibrillary tangles in AD, and it is also the key to a variety of neuronal survival signals to interfere with neuronal apoptosis (Yang et al., 2018b; Chang et al., 2019; Shi et al., 2019). Recent studies have identified GSK-3β as a potential therapeutic target in Alzheimer’s disease (Shi et al., 2019). Cornel Iridoid Glycoside, a promising agent for AD therapy, inhibits tau hyperphosphorylation *via* regulating cross-talk between GSK-3β and PP2A signaling (Yang et al., 2018a). Osthole decreases tau protein phosphorylation *via* PI3K/AKT/GSK-3β signaling pathway in AD (Yao et al., 2019). Consistent with previous studies, our results showed that AMI inhibits the expression of the tau upstream kinase GSK-3β. In addition, tau protein kinase II (TPKII) formed by a complex containing two subunits of Cyclin-dependent Kinase 5 (CDK5) and p35 can synergistically increase the efficiency of GSK-3β phosphorylation of tau protein (Xiao et al., 2018; Giannopoulos et al., 2019). Therefore, we need to investigate the expression of tau protein kinase II (TPKII) in the next experiment.

This study also suggested that AMI has a therapeutic effect on the PD animal model *in vivo*. Continuous injection high-dose MPTP for 5 days was used to prepare a PD mice model, and levodopa was used as a positive control. The dopamine neurons were destroyed by MPTP, and dopamine levels were decreased in mice. Dopamine was not replenished in a short time, so the behavioral abnormalities of MPTP group became serious, but AMI protected the dopamine neurons of mice from MPTP injury, while L-DOPA was supplemented with dopamine, which also improved Parkinson’s behavioral abnormalities (Thomas et al., 2019). On the contrary, the TH immunohistochemistry results shown that number of DA neurons in the L-DOPA group was not significantly higher than that in the MPTP group, which further illustrates the current major drawbacks of L-DOPA treatment with PD, these drugs can only improve symptoms, but cann’t effectively delay the progression of the disease and prevent the apoptosis of dopamine neurons. In addition, with the long-term application of L-DOPA, its efficacy gradually decreased, most patients will tolerate complications, such as, dyskinesia or mental disorders (Wichmann, 2019).

The maintenance of the density of striatum dendritic spines can delay the exacerbation of PD, prolong the use time of levodopa and improve its therapeutic effect (Deutch et al., 2007). Golgi staining was used to observe the morphology of neurons (Czechowska et al., 2019). AMI significantly increases the density of neuronal dendrites in the substantia nigra of mice. Notely, the increase of dendritic spine density of striatum can compensate for PD animal striatum DA level reduction and promote the recovery of their behavioral function (Kim et al., 2013). Our behavioral experiment results are consistent with literature reports. In general, AMI has neuroprotective and neurotrophic effects. On the one hand, it protects the integrity of dendritic spines, on the other hand, AMI saves the loss of dopamine neurons, and effectively improves the behavioral abnormalities of C57/BL mice.

## Data Availability Statement

The datasets generated for this study are available on request to the corresponding author.

## Ethics Statement

The animal study was reviewed and approved by the Institutional Animal Care and Use Committee of Southern Medical University (Guangzhou, Guangdong, China).

## Author Contributions

ZM conducted the experimental work, analysis and interpretation of the data. ZW-t conceived and drafted the manuscript. WW-y, XJ-p and WH-t designed the study and critically revised the manuscript. Other authors were involved in data collection and checked the manuscript. All authors contributed to the article and approved the submitted version.

## Conflict of Interest

The authors declare that the research was conducted in the absence of any commercial or financial relationships that could be construed as a potential conflict of interest.
